# *In vitro* and *in silico* evaluation of *Andrographis paniculata* ethanolic crude extracts on fatty acid synthase expression on breast cancer cells

**DOI:** 10.37796/2211-8039.1444

**Published:** 2024-06-01

**Authors:** Nur Amanina Johari, Nur Anisa Sapi’i, Alvin Lu Jiunn Hieng, Nurriza Ab Latif, Syazwani Itri Amran, Rosnani Hasham, Khairunadwa Jemon

**Affiliations:** a Department of Biosciences, Faculty of Science, Universiti Teknologi Malaysia, 81310 Skudai, Johor, Malaysia; b Institute of Bioproduct Development, Universiti Teknologi Malaysia, 81310 Skudai, Johor, Malaysia; c Department of Bioprocess and Polymer Engineering, School of Chemical and Energy Engineering, Faculty of Engineering, Universiti Teknologi Malaysia, 81310 Skudai, Johor, Malaysia

**Keywords:** *Andrographis paniculata*, Apoptosis, Breast cancer, Fatty acid synthase, Molecular docking

## Abstract

**Background:**

Fatty acid synthase (FASN), a key rate-limiting enzyme in the fatty acid biosynthesis pathway has been identified to be overexpressed in breast cancer. This overexpression has been affiliated with poor prognosis and resistance to chemotherapeutics. Consequently, FASN has come into focus as an appealing potential target for breast cancer treatment. Available FASN inhibitors, however, are unstable and have been correlated with adverse side effects.

**Objective:**

This present study aims to investigate the potential of *Andrographis paniculata* ethanolic crude extract (AP) as a potent FASN inhibitor in breast cancer cells.

**Materials & methods:**

This study used MTT assay and flow cytometry analysis to measure cell viability and apoptosis following AP treatment (0–500 μg/mL). Furthermore, FASN protein expression was evaluated using immunocytochemistry whereas lipid droplet formation was quantified using Oil Red O staining. Literature-based identified AP phytochemicals were subjected to the prediction of molecular docking and ADMET properties.

**Results:**

This study demonstrated that AP significantly reduced cell viability while inducing apoptosis in breast cancer cells. In addition, for the first time, exposure to AP was demonstrated to drastically reduce intracellular FASN protein expression and lipid droplet accumulation in EMT6 and MCF-7 breast cancer cells. Docking simulation analysis demonstrated AP phytochemicals may have exerted an inhibitory effect by targeting the FASN Thioesterase (TE) domain similarly to the known FASN inhibitor, Orlistat. Moreover, all AP phytochemicals also possessed drug-likeness properties which are in accordance with Lipinski’s rule of five.

**Conclusions:**

These results highlight the potential of *A. paniculata* ethanolic crude extract as a FASN inhibitor and hence might have the potential to be further developed as a potent chemotherapeutic drug for breast cancer treatment.

## 1. Introduction

Breast cancer is the most prevalent malignant neoplasm that affects women all over the world [1,2]. Despite significant advancements in contemporary medicine and the development of innovative therapeutic strategies, cancer recurrences continue to be a prominent cause of morbidity and mortality in women with breast cancer [3,4]. Deregulated lipid metabolism is one of the cancer hallmarks with increased fatty acid biosynthesis being one of the most notable abnormalities [5,6]. In contrast to normal cells which obtain fatty acids through dietary sources, the activation of the *de novo* fatty acid biosynthesis pathway allows tumour cells to create fatty acids to promote and sustain a higher rate of growth and proliferation [7–9]. In proliferating cancer cells, fatty acids and their metabolic intermediaries are utilized for ATP synthesis through mitochondrial β-oxidation, cell signaling via palmitoylation of oncogenic proteins, or maintenance of membrane integrity [10–15].

Fatty acid synthase (FASN) is an important enzyme that catalyzes the production of long-chain fatty acids, palmitate, from acetyl-CoA and malonyl-CoA in the presence of NADPH in the *de novo* fatty acid synthesis pathway [16,17]. The overexpression of FASN has been discovered in a wide variety of human malignancies including breast cancer [8,18] and it is correlated to rapid tumor progression, poor prognosis, and high risk of death. In normal cells, however, this particular enzyme is not expressed or expressed at a very low level [19,20]. The differential expression of FASN between cancer cells and normal cells makes it a potential diagnostic tumor marker. Furthermore, because of its specific cytotoxicity to cancer cells, FASN has been identified as an intriguing potential target for cancer therapy. This enzyme inhibition has been found to significantly impede tumor development and survival, suppress aggressiveness and metastasis, and promote cancer cell apoptosis while exerting minimal effect on normal cells [17,21]. However, effective FASN inhibitors for cancer treatment remain inadequate. Natural products (for example, green tea-derived epigallocatechin-3-gallate) [7,22,23] and synthetically manufactured compounds (for example, orlistat [24–26], cerulenin and C75 [17,27,28]) have been investigated as potential inhibitors of FASN for cancer treatment. There is an ongoing phase II clinical trial that investigates the efficacy of FASN inhibitor TVB-2640 and trastuzumab in combination with either paclitaxel or endocrine treatment with an aromatase inhibitor to treat metastasized HER2-positive breast cancer patients. Despite that, none of these FASN inhibitor candidates have been clinically approved for cancer treatment due to poor stability, bioavailability, and the occurrence of adverse side effects [17,27]. Furthermore, the development of FASN inhibitors continued to slow down because enzyme inhibition has been associated with several safety issues, including the buildup of metabolic intermediates and organ-specific toxicity [29]. Hence, the development of a pharmaceutical-grade FASN inhibitor from natural resources to address this metabolic dysregulation offers great promise for breast cancer treatment.

Medicinal plants used in conventional and complementary medicines are continuing to be investigated for malignancy treatment and chemoprevention. *Andrographis paniculata*, commonly known as “Hempedu Bumi” in Malaysia, is a popular traditional medicine used extensively across Asia [30–32]. This medicinal plant contains diverse phytochemicals including labdane diterpenoid lactones, flavonoids and miscellaneous phytochemicals that possess a wide spectrum of pharmacological properties and biological applications, such as anti-microbial [33,34], antioxidant [35,36], antiangiogenic [37,38], anti-diabetic [39], anti-inflammatory and immunostimulatory [40–42]. Owing to its high antioxidant capacity, *A. paniculata* extract has also recently been exploited to synthesize different types of nanoparticles which assists in the removal of potentially harmful by-products while also aiding in nanoparticle fine-tuning [43–45]. Aside from that, several studies have demonstrated the anticancer properties of *A. paniculata*, suggesting its intriguing potential as a prospective chemotherapeutic agent [46,47]. *A. paniculata* crude extracts and its phytochemicals have been shown to inhibit cell proliferation and cause apoptosis in various cancer types including breast [48,49], neuroblastoma [50], and esophageal cancer [51]. In addition, *A. paniculata* and its phytochemicals are already known for their antiadipogenic and hypolipidemia effects in macrophages [52] and adipocytes [53]. Nonetheless, there have been no findings on the probable mechanism behind the effect of AP on fatty acid metabolism in breast cancer. Therefore, the present study was undertaken to investigate the underlying anticancer mechanism of *A. paniculata* ethanolic crude extract on FASN expression in breast tumor cells, for the first time.

## 2. Materials & methods

### 2.1. Preparation of plant extract and stock solution

The dried leaves of *A. paniculata* were grounded into a powdered form before being extracted using the ultrasonication-assisted extraction (UAE) technique. In brief, 2.5 g of powdered *A. paniculata* leaves were extracted in 50 mL ethanol (HmbG, Hamburg, Germany). The solution was then sonicated for 30 min at 30°C using a water sonicator (Hinotek, Ningbo, China). The solution was filtered and collected using filter paper (Whatman, Wales, UK) before drying in an oven at 60 °C for 24 h. The dried extract was stored at 4 °C until future usage. To prepare 1000 μg/ml of AP stock solution, 1 mg of dried extract was dissolved in 0.25% Tween 20 (SYSTERM, Selangor, Malaysia) followed by the addition of 0.25% dimethyl sulfoxide (DMSO) (Merck Millipore, Burlington, MA, USA). Then, fresh complete media was pipetted to make up 0.5% Tween 20 and DMSO in the final volume. After that, the solution was resuspended and vortexed until it thoroughly dissolved before being filtered through a syringe filter [54].

### 2.2. Cell culture

Breast cancer cells, EMT6, MCF-7, and MDA-MB-231 were maintained and cultured in Waymouth medium, RPMI 1640 medium, and Dulbecco’s Modified Eagle’s Medium/F-12 (Life Technologies, Carlsbad, CA, USA), respectively. Human skin fibroblast cells (HSF1184) and human epidermal keratinocytes (HaCaT) were used as controls. HSF1184 and HaCat cells were maintained in Dulbecco’s modified Eagle medium (DMEM). All media were supplemented with 10% heat-inactivated fetal bovine serum (FBS) (Gibco, Billings, MT, USA) and 1% of Penicillin-Streptomycin (Gibco, Billings, MT, USA). The cells were maintained at 37 °C in an incubator with 5% CO_2_ (Esco, Singapore).

### 2.3. Cell viability assay

EMT6, MCF-7, MDA-MB-231, HSF1184, and HaCaT cells were seeded at 1 × 10^5^ cells/well into a 96-well plate and then treated with the AP ethanolic crude extract in various concentrations (7.81–500 μg/ml) for 24 h and 48 h, while control cells were left untreated. Following that, 0.5 mg/ml MTT (Thermo Fisher Scientific, Waltham, MA, USA) solution was added to each well and incubated for 3 h at 37 °C. The supernatant was then aspirated, 100 μl of 100% DMSO was added into each well before the plate was left for 20 min. Finally, the absorbance was measured at 570 nm by using a Spectro Star Nano microplate reader (BMG Labtech, Ortenberg, Germany) [55].

### 2.4. Apoptosis assay

Annexin-V FITC/PI Apoptosis Detection Kit (Invitrogen, Waltham, MA, USA) was used to detect cell apoptosis according to the manufacturer’s protocol. Firstly, the cells were seeded at 2 × 10^6^ cells/well in a 6-well plate and treated with varying concentrations of AP ethanolic crude extract for 48 h. Untreated cells served as the negative control while 20 nM of Taxol (Sigma-Aldrich, St. Louis, MO, USA) was used as the positive control. The cells were then extracted from the plate, rinsed once in cold phosphate-buffered saline (PBS) (Oxoid, Hampshire, UK), and resuspended in 195 μl 1 × binding buffer. The cell suspension was then incubated for 10 min in the dark at room temperature with 5 μl of Annexin-V FITC. The cells were washed with 1 × binding buffer before being resuspended in 190 μl of 1 × binding buffer. The cell suspension was then treated with 10 μl of Propidium Iodide (PI) and maintained on ice in the dark. Flow cytometry analysis was performed by using BD FACSVerse™ flow cytometer (Becton Dickinson, Franklin Lakes, NJ, USA) before being analyzed by using the FlowJo software program version 10.6.1 (Treestar Inc, Ashland, OR, USA).

### 2.5. Immunocytochemical analysis

Cells were seeded on a 13 mm glass coverslip before being administered for 48 h with AP. The untreated cell served as a control. The cells were fixed with 1% paraformaldehyde (PFA) (Sigma-Aldrich, St. Louis, MO, USA) for 20 min. After that, the coverslips were rinsed twice with PBS. The cells were further permeabilized in 0.1% of Triton X-100 (Sigma-Aldrich, St. Louis, MO, USA) at room temperature in the dark for 30 min. After two washes with PBD, the cells were then incubated with 100 μL of FASN goat anti-mouse IgG antibody (Santa Cruz Biotechnology, Dallas, TX, USA) diluted in 7% FBS overnight at 4 °C. Next, the cells were rinsed with two PBS washes before being incubated with 100 μL Alexa Flour 488 goat anti-mouse IgG (Thermo Fisher Scientific, Waltham, MA, USA) diluted in 7% FBS at 4 °C for 3 h. After repeated washes with PBS, the coverslips were counterstained with two drops of nuclear probe (Life Technologies, Carlsbad, CA, USA) and let to sit at room temperature in the dark for 10 min. Following two PBS rinses, the coverslips were mounted with DPX mounting medium (Sigma-Aldrich, St. Louis, MO, USA) on the glass slide. The slides were visualized under a fluorescent microscope (Nikon, Tokyo, Japan).

### 2.6. Oil Red O staining

Breast cancer cells were stained with Oil Red O (Sigma-Aldrich, St. Louis, MO, USA) to determine their intracellular lipid content following treatment for 48 h with AP. In brief, treated cells were incubated for 30 min with 10% Neutral Buffered Formalin (Leica, Wetzlar, Germany) at room temperature. The cells were then washed once with 60% isopropanol (Supelco, Bellefonte, PA, USA) and stained for 15 min with 0.3% Oil Red O Working Solution before being thoroughly rinsed with ddH_2_O for the removal of unbound dye. The cells need to be submerged with ddH_2_O while being observed under a microscope. Lipid droplets will appear red. Lipid content was quantified by eluting Oil Red O dye from the stained cells with 100% isopropanol and measuring light absorbance at 510 nm using a Spectro Star Nano microplate reader [56].

### 2.7. Molecular docking

The docking study was carried out within the Thioesterase domain of the active site of the FASN (PDB code: 2PX6), retrieved from the Protein Data Bank (PDB) database using AutoDock Tools (ADT) version 1.5.6 (Scripps Research, San Diego, CA, USA). The apo-protein structure was prepared by removing the co-crystal ligand and water molecules followed by adding the Gasteiger charges and merging the non-polar hydrogen atoms. A grid box of 40 × 40 × 40 was built and centered on the ligand with a spacing of 0.375 A. The ligand binding coordinates (x = 12.75, y = −4.297, z = 25.282) were determined through the control docking procedure. The chemical structure of AP phytochemicals (andrographolide, isoandrographolide, neoandrographolide, and 14-deoxy-11,12-didehydroandrogapholide) was retrieved from the PubChem database were subjected to 50 dependent docking runs. The best docking conformations were selected based on the free binding energy values and superimposition with the crystal structure. Protein/ligand interactions were determined using Discovery Studio Visualizer 4.0 (BIOVIA, San Diego, CA, USA).

### 2.8. ADMET analysis

Prediction of the drug-likeness and pharmacokinetic properties; ADMET (Absorption, Distribution, Metabolism, Excretion and Toxicity) of the selected phytochemicals of AP (andrographolide, isoandrographolide, neoandrographolide and 14-deoxy-11,12-didehydroandrographolide) were performed using SWISS-ADME and pkCSM web server. The SMILES code of selected phytochemicals was obtained from PubChem and submitted to SWISS-ADME and pkCSM online tools. Parameters such as the number of hydrogen bond donors (HBD), hydrogen bond acceptors (HBA), total polar surface area (TPSA) value, lipophilicity, water solubility, P-glycoprotein (P-gp) substrate, gastrointestinal (GI) absorption, blood–brain barrier (BBB) permeability, and cytochrome P450 inhibitors were evaluated.

### 2.9. Statistical analysis

Shapiro-Wilk test was used to determine the normality of the data distribution and a two-sample t-test was performed to evaluate the comparisons across experimental groups. The mean of triplications with standard error mean (±SEM) was used to assess the data. All statistical analyses were carried out using GraphPad Prism version 9.0 (GraphPad Software, La Jolla, CA, USA). *P* values of ≤ 0.05 were deemed statistically significant.

## 3. Results

### 3.1. AP inhibited breast cancer cells proliferation

The effect of various concentrations of AP (7.81, 15.63, 31.25, 62.5, 125, 250, and 500 μg/mL) for 24 and 48 h on breast cancer (EMT6, MDA-MB-231, and MCF-7) and normal (HSF1184 and HaCaT) cells viability were measured by MTT assay. As can be seen in Table 1, AP reduced cell viability in a concentration- and time-dependent inhibition on breast cancer cell lines. MCF-7 induced significant cytotoxicity at IC_50_ value of 35.19 μg/ml followed by MDA-MB-231 (58.8 μg/ml) and EMT6 (74.8 μg/ml) after 24 h of AP treatment. The IC_50_ values of treatment on MCF-7, MDA-MB-231, and EMT6 were found to be 29.35 μg/mL, 27.08 μg/mL, and 45.15 μg/mL, respectively. In contrast, the IC_50_ value of AP on normal cells HSF-1184 and HaCaT were observed thrice greater than on breast cancer cell lines after 24 h and 48 h treatment, indicating that AP had no substantial cytotoxic effect on normal cells. These findings showed that AP had a significant selective inhibitory effect on the proliferation of breast cancer cell lines causing a minimal cytotoxicity in normal cell lines.

### 3.2. APcaused breast cancer cells to undergo apoptosis

**Fig. 1:**
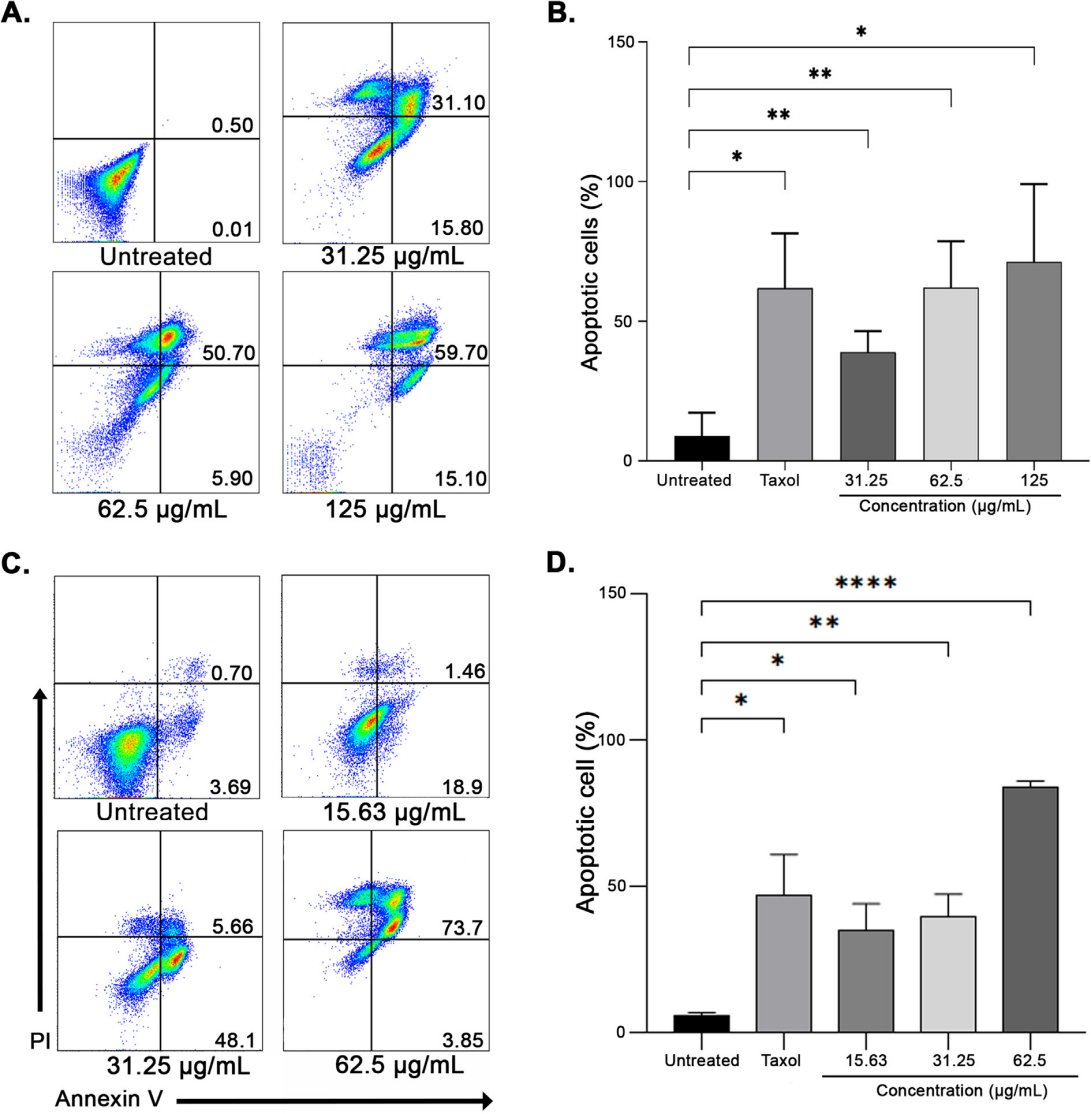
AP-induced apoptosis in breast cancer cells. (A) EMT6 and (C) MCF-7 cells were treated with AP at the indicated doses for 48 h, labelled with Annexin V-FITC and PI, and then analyzed by flow cytometer. The percentage of apoptotic cells (mean ± SEM) after treatment for EMT6 (C) and MCF-7 (D). Data are shown from three independent experiments (n = 3). *P < 0.05 and **P < 0.01, and ****P < 0.0001 (Two sample t-test).

The pro-apoptotic effect of AP in MCF-7 and EMT6 breast cancer cells was evaluated by using the Annexin V/PI double staining and flow cytometry analysis. Treatment of EMT6 cells for 48 h with AP demonstrated that the treatment increased the percentage of apoptotic cells in a concentration-dependent manner (Fig. 1A,B). Increased cell death also is similarly associated with increasing AP concentration on MCF-7 cells as demonstrated in Fig. 1C,D. AP administration resulted in a dosage-dependent and statistically significant increase of apoptotic cells in both EMT6 and MCF-7 cell lines, which was consistent with the corresponding decrease in cell proliferation described in the previous section.

### 3.3. AP reduced expression of FASN in EMT6 and MCF-7 cells

**Fig. 2:**
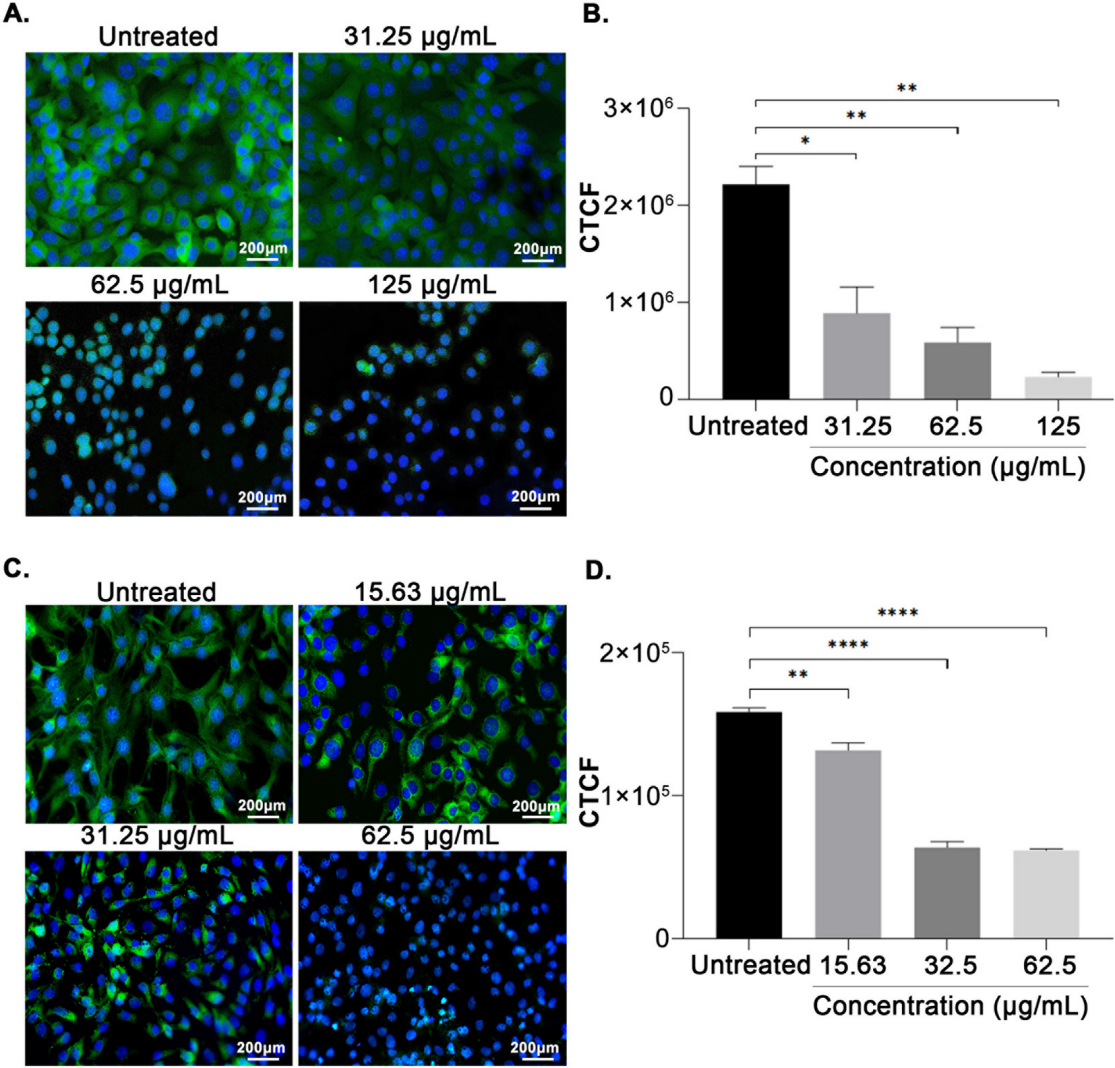
Intracellular expression of FASN in breast cancer cells. Fluorescence micrographs showing the expression of FASN on EMT6 (A) and MCF-7 (B). The mean corrected total cell fluorescence of FASN expression (mean ± SEM) for EMT6 (C) and MCF-7 (D) is shown from three independent experiments (n = 3). *P < 0.05, **P < 0.01, ***P < 0.001 and ****P < 0.0001 (Mann Whitney test).

To identify the potential targets via which AP may exert its inhibitory effect on MCF-7 and EMT6 breast cancer cell lines, the profile of the FASN expression was analyzed after treating the cells with AP using immunocytochemical analysis. Fluorescence microscopic images of FASN proteins in AP-treated EMT6 and MCF-7 cells are shown in Fig. 2A and C. As illustrated in Fig. 2B, AP decreased FASN protein expression in EMT6 after 48 h of treatment. Immunofluorescence quantitation using corrected total cell fluorescence (CTCF) also demonstrated a statistically significant reduction in the FASN protein expression in AP-treated MCF-7 cells in comparison with corresponding untreated cells (Fig. 2D).

### 3.4. AP treatment decreased intracellular levels of lipid droplets

**Fig. 3:**
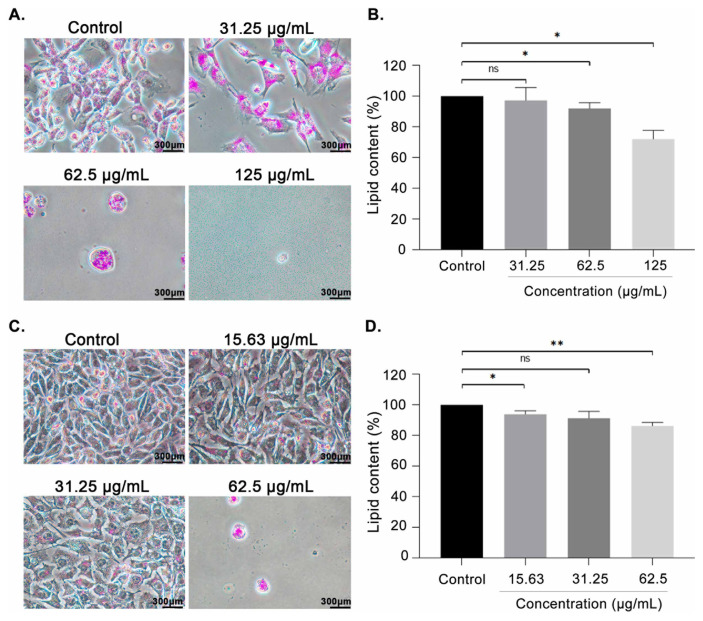
AP decreased lipid droplet accumulation in breast cancer cells. Oil Red O staining was used to visualize the lipid droplet changes in EMT6 (A) and MCF-7 (B) cells treated with AP or left untreated. The percentage of lipid content (mean ± SEM) for EMT6 (C) and MCF-7 (D) is shown from three independent experiments (n = 3). ns: non-significant, *P < 0.05, and **P < 0.01 (Two sample t-test).

Previous findings demonstrated that AP inhibited fatty acid synthesis in breast cancer cells, which is a necessary component for lipid droplets. Therefore, to further demonstrate the FASN inhibitory effect of AP, EMT6, and MCF-7 breast cancer cells were treated with the crude extract for 48 h, and lipid droplets accumulation was assessed by Oil Red O staining. As expected, compared with the control group, Oil Red O results demonstrated that FASN inhibition by AP significantly decreased lipid contents dose-dependently, showing fewer lipid droplets in both cell lines (Fig. 3).

### 3.5. Docking analysis of AP phytochemicals with FASN

**Fig. 4:**
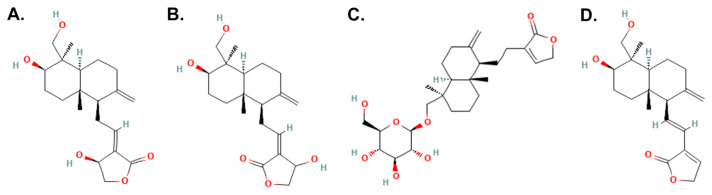
Chemical structures of selected compounds: (A) Andrographolide, (B) Isoandrographolide, (C) Neoandrographolide, and (D) 14-deoxy-11,12-didehydroandrographolide from A. paniculata ethanolic crude extract.

**Fig. 5:**
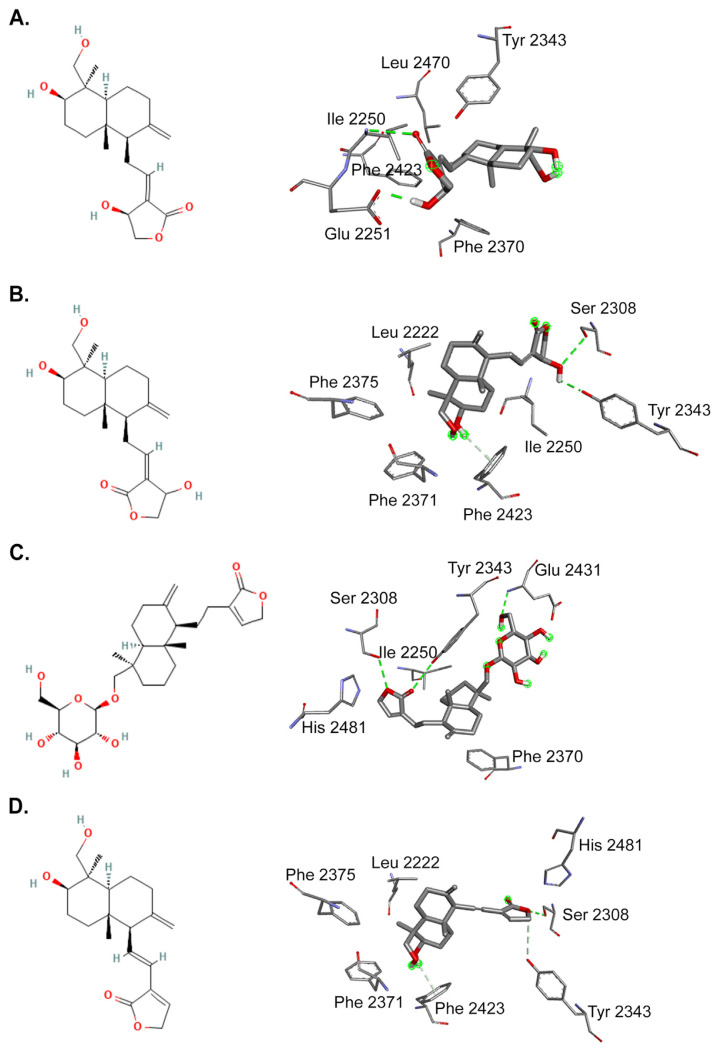
The 3-D (right) and 2-D (left) representations of protein-ligand interaction between (A) Andrographolide, (B) Isoandrographolide, (C) Neoandrographolide and (D) 14-deoxy-11,12-didehydroandrographolide and Thioesterase domain of FASN with accession code 2PX6.

To gain more insight into the inhibitory action of *A. paniculata* in binding to FASN, molecular docking was used to predict the inhibition activity of selected phytochemicals on FASN Thioesterase (TE) domain. Four AP phytochemicals; andrographolide, isoandrographolide, neoandrographolide, and 14-deoxy-11,12-didehydroandrographolide identified from published literatures [34,57–60] (Fig. 4) demonstrated higher binding affinity to FASN TE in comparison to the co-crystal ligand, orlistat (Table 2). 14-deoxy-11,12-didehydroandrographolide and isoandrographolide showed the highest affinity with a free binding energy value of −7.04 kcal/mol and −7.00 kcal/mol respectively followed by andrographolide (−6.57 kcal/mol) and neoandrographolide (−6.37 kcal/mol) whereas orlistat, a US FDA approved drug for obesity showed lower binding affinity to FASN (−5.34 kcal/mol). The interactions of the phytochemicals with the TE domain are illustrated in Fig. 5. The andrographolide- and 14-deoxy-11,12-didehydroandrographolide-TE established three hydrogen bonds with Ser2308, Try2343, and Phe2423 while isoandrographolide only formed two hydrogen bonds with Ser2308 and Tyr2343. On the other hand, neoandrographolide formed three hydrogen bonds with Ser2308, Glu2431, and Tyr2343. These phytochemicals made a similar interaction as orlistat through hydrogen bonds with Ser2308 and demonstrated additional hydrogen bonds with Tyr2343 and Phe2423 in the binding site.

### 3.6. ADMET analysis

The safety and efficacy of drug candidates can be predicted by evaluating the drug-likeness and pharmacokinetic properties. Hence, drug-likeness, bioavailability, absorption, distribution, metabolism, excretion, and toxicity (ADMET) properties of tested phytochemicals were predicted using SwissADME and pkCSM tools. According to Lipinski’s rule, the compounds with zero or single violations of criteria were considered to have higher bioavailability in the metabolic process and were more likely to be administered as oral drugs [61]. The parameters in Lipinski’s rule comprised of the (1) molecular mass (MW) of the compound, not more than 500 g/mol; (2) no more than 5 hydrogen bond donors (NH or OH); (3) no more than 10 hydrogen bond acceptors (N or O); and (4) the octanol-water partition coefficient (log P) value not greater than 5. The predicted results showed that selected phytochemical compounds showed good drug-likeness properties indicated by Lipinski’s Rule of five and Veber by having a molecular weight less than 500 g/mol, number of hydrogen bond donor (HBD) less than 5 and hydrogen bond acceptors (HBA) less than 10, number of rotational bonds less than 10, log P less between 0 and 5 and TPSA value less than 140 A which refer to Lipinski properties only. Based on the prediction outcomes in Table 3, all compounds were predicted to have the potential to be oral drug candidates as they were estimated to have high gastrointestinal (GI) absorption potency. All four AP phytochemicals also demonstrated acceptable safety profiles on the heart, liver, and skin as well as not carcinogenic except for neoandrographolide since it might induce liver injury. According to the ADMET results, the AP phytochemicals have favorable pharmacokinetic and drug-likeness features with minimal toxicity.

## 4. Discussion

Abnormal lipid metabolism has been widely identified as a diagnostic marker of breast cancer [13,62]. Continuous lipogenesis fuels the rapid proliferation of cancer cells by providing them with energy, protein posttranslational modifications, membrane building blocks, signaling lipid molecules, and lipid molecules [63]. Overexpression of FASN was shown to promote cell growth while impairing programmed cell death in a wide range of cancer cell types [17,22]. AP and its bioactive phytochemicals have been demonstrated to exhibit anticancer properties towards breast cancer cells by their ability to induce cytotoxic [48], pro-apoptotic [64,65], antiangiogenic [66–68], and antimetastatic effects, however, it is not yet known whether AP is capable of inducing cell apoptosis by interfering with lipogenesis in breast cancer cells. The present study investigates the pharmacological impact of AP on fatty acid synthase in both human and murine breast cancer cells. It is expected that inhibiting the FASN enzyme by AP will decrease the survivability of breast cancer cells given the significance of the protein in providing essential lipids for oncogenic processes. It has been demonstrated that AP inhibited breast cancer cells proliferation (Table 1) and induced apoptosis in a concentration-dependent manner compared with the untreated cells after 48 h of treatment (Fig. 1). Other studies have also highlighted the FASN inhibitory effects of medicinal plants and its substantial downstream anticancer effects (Table 4). The downregulation of FASN by plant extracts has been closely associated with the induction of apoptosis and cytotoxicity in breast cancer, in line with the observation found in the present study (Fig. 2). In both EMT6 and MCF-7 cells, the outcome was followed by a decrease in lipid droplet levels (Fig. 3). The results revealed for the first time that AP potently inhibited FASN in breast cancer cells, as evidenced by its ability to reduce lipid accumulation and inhibiting FASN Thioesterase (TE) domain (Fig. 5).

To better understand the mechanism by which AP induced apoptosis, the expression of FASN protein in treated cells was examined. Our results in Fig. 2 showed that AP could induce apoptosis in breast cancer cells by inhibiting the lipogenic protein. The results from this study were in line with the findings of Benjamin *et al.* [69] who discovered that inhibition of FASN decreased the synthesis of oncogenic signaling lipid complexes such as diacylglycerol (DAGs) which in turn reduced the activation of protein kinase C (PKC) stimulation. The decrease in PKC causes the pro-apoptotic factor, Bax to be dephosphorylated and activated, which causes the cancer cells to undergo apoptosis. Additionally, it is also proposed that FASN blockade is likely to increase the fatty acid synthesis substrate levels causing cytotoxicity and promoting apoptosis. A study conducted by Kuhajda et al. (2001) found that FASN inhibition leads to fatty acid synthesis substrate, malonyl-CoA levels accumulation which in turn inhibit carnitine palmitoyltransferase (CPT-1) and transfer of palmitate to mitochondria, preventing fatty acid beta-oxidation and causing energy starvation in cancer cells. Following pharmacological FASN inhibition, elevated levels of Bax and cytochrome c were detected in multiple tumor cell lines, indicating mitochondrial participation in apoptosis. These findings may be utilized to explain how apoptosis is induced by inhibiting FASN in AP-treated cancer cells [24,56,70].

Since FASN is essential for endogenous fatty acid production, its expression in breast cancer cells was crucial for intracellular lipid production. It has been well established that lipid accumulation is closely related to carcinogenesis, aggressiveness, and treatment resistance in cancer [23]. Oil Red O results in Fig. 3 shared similarities with Wang et al. (2012) findings which demonstrated that FASN inhibition can induce breast cancer cell apoptosis by decreasing lipid droplet accumulation [71]. Based on the phenomenon, the inhibition of palmitate synthesis, which is essential for membrane synthesis, protein-protein signaling, and ATP production is suspected to cause cell apoptosis. Depletion of palmitate is expected to cause reorganization of membrane architecture and disruption of lipid raft domains associated with oncogenic signaling complexes. By disrupting membrane structure, FASN inhibition may disable signal transduction networks and biological processes needed for cell growth, proliferation, and response to cellular stress [72]. Cancer cells showed a preference for the utilization of endogenous fatty acids and fatty acid-derived lipids to support malignant processes, so the limitation of the substrates leads to disruption in proper protein trafficking and localization as well as membrane organization and architecture. The addition of palmitate has been found to completely or partially rescue cancer cells from induced death by FASN inhibition [73]. The present study hypothesized that a deficiency in fatty acids caused by FASN expression inhibition was the underlying reason of reduced lipid droplet formation and increased cell death in AP-treated cells.

Furthermore, molecular docking simulation was performed to further screen potential anticancer phytochemicals in AP that may exert their pharmacological effects by binding FASN Thioesterase domain (FASN TE) (Fig. 4). Interestingly, AP is suggested to inhibit lipid droplet accumulation by binding to the active site of FASN TE, preventing the release of palmitate from the protein (Fig. 5). According to the docking results, AP phytochemicals have similar affinity for the FASN TE binding site as palmitate and orlistat, a FASN inhibitor. Compared to other FASN catalytic domains, FASN TE is a particularly promising target for breast cancer therapy because it is involved in determining the length and terminating palmitate, long chain fatty acids precursor [74]. The palmitate then will be used as a structural component for the new plasma membrane and to generate the required energy for breast cancer progression via fatty acid β-oxidation. Carboxyl group of palmitate binds into the ligand-binding pocket that encompasses residues important for the catalytic function of the TE domain such as the catalytic triad, Ser2308, His2481, and Asp2338 [75]. Orlistat, an FASN inhibitor, interferes with FASN by interacting at the TE domain preventing the enzyme from hydrolyzing palmitate causing cancer cells to undergo cell death [24,25,76]. By binding to the catalytic triad Ser-His-Asp residues in the active site (Fig. 5), AP phytochemicals significantly inhibit fatty acid synthesis by blocking the action of palmitate binding and termination by FASN TE which was reflected by depleted lipid droplet formation (Fig. 3). These interactions may contribute to downregulation of FASN catalytic activity and lipid droplets accumulation which resulted in inhibition of breast cancer cells growth. Based on the above reasons, we suggest that all phytochemicals of *A. paniculata* showed competent docking energy values and have similar interaction behavior as Orlistat, a FASN inhibitor that binds to the binding site of the FASN TE domain. Further, *in vitro* investigations on inhibition of FASN TE by the selected AP phytochemicals should be conducted to confirm the *in silico* results.

## 5. Conclusion

In summary, the present study revealed that AP treatment could reduce cell viability and induce cell apoptosis in EMT6 and MCF-7 breast cancer cells by inhibiting FASN protein expression and reducing lipid droplet accumulation. Additionally, all selected AP phytochemicals have also shown promising binding affinity to FASN TE in comparison with Orlistat while possessing acceptable ADMET properties. Comprehensive *in vitro* investigations are required to isolate and identify bioactive phytochemicals in AP that inhibit FASN in breast cancer cells. Taken together, these results from this study provide extended insight into AP’s novel mechanism of action and possible metabolic therapeutic interventions in treating breast cancer.

## Figures and Tables

**Table 1:** The IC_50_ (μg/mL) value of A. paniculata in breast cancer cells and normal cells. The data are presented as the mean ± SD of at least the threeindependent experiment (n = 3).

Time (h)	Breast cancer cells			Normal cells	
	
	EMT6	MDA-MB-231	MCF-7	HSF 1184	HaCaT
24	74.8 ± 9.1	58.8 ± 16.1	35.2 ± 3.8	173.4 ± 6.7	170.4 ± 38.4
48	45.2 ± 5.1	27.1 ± 14.9	29.4 ± 17.9	105.2 ± 16.7	105.8 ± 12.7

**Table 2:** The binding affinity of A. paniculata towards FASN.

Ligand	Free Binding energy (kcal/mol)	Estimated inhibition constant (μM)
Orlistat	−5.34	120.87
Neoandrographolide	−6.37	21.34
Andrographolide	−6.57	15.22
Isoandrographolide	−7.00	7.38
14-deoxy-11,12-didehydroandrographolide	−7.04	6.91

**Table 3:** Physiochemical and pharmacokinetic properties of phytochemicals in A. paniculata by SwissADME and pKCSM analysis.

		Phytochemicals			
		Andrographolide	Isoandrographolide	Neoandrographolide	14-deoxy-11,12-didehydroandrographolide
**Physiochemical properties**	MW (g/mol)	350.45	350.45	480.59	332.43
	Fsp^3^	0.75	0.75	0.81	0.65
	RB	3	3	7	3
	HBA	5	5	8	4
	HBD	3	3	4	2
	MR	95.21	95.21	125.27	93.58
	TPSA (Å^2^)	86.99	86.99	125.68	66.76
**Lipophilicity Drug-likeness**	logP	2.30	2.30	2.31	3.02
	Lipinski	No violation	No violation	No violation	No violation
	Ghose	No violation	No violation	2 violations (MW > 480, #atoms >70)	No violation
	Veber	No violation	No violation	No violation	No violation
	Egan	No violation	No violation	No violation	No violation
	Muegge	No violation	No violation	No violation	No violation
**Water Solubility**	ESOL	−3.18	−3.18	−4.01	−3.74
	Log W	2.34e-01 mg/ml; 6.67e-04 mol/l	1.55e-01 mg/ml; 4.42e-04 mol/l	4.65e-02 mg/ml; 9.67e-05 mol/l	6.08e-02 mg/ml; 1.83e-04 mol/l
	Class	Soluble	Soluble	Moderately soluble	Soluble
**Pharmacokinetics**	GI absorption	High	High	High	High
	BBB permeant	No	Yes	No	Yes
	P-gp substrate	Yes	Yes	Yes	Yes
	CYP1A2 inhibitor	No	No	No	No
	CYP2C19 inhibitor	No	No	No	No
	CYP2C9 inhibitor	No	No	No	Yes
	CYP2D6 inhibitor	No	No	No	No
	CYP3A4 inhibitor	No	No	Yes	Yes
	Log *K_p_* (cm/s)	−6.90	−6.78	−7.36	−6.03
	Bioavailability	0.55	0.55	0.55	0.55
**Toxicity**	Carcinogenicity (AMES Toxicity)	No	No	No	No
	Cardiovascular toxicity (hERG I Inhibitor)	No	No	No	No
	Hepatotoxicity	No	No	Yes	No
	Skin Sensitization	No	No	No	No

Molecular weight: MW, topological polar surface area: TPSA, Molar Refractivity: MR, fraction of sp^3^ carbon atoms: Fsp^3^, HBD: hydrogen bonds donor, HBA: hydrogen bond acceptor, RB: rotatable bonds, LogP values: indicator of Lipophilicity, ESOL: aqueous solubility parameter, GI absorption: gastrointestinal absorption, BBB: blood–brain barrier, P-gp: P-glycoprotein, Log *K_p_*: skin permeation, hERG: human Ether-à-go-go-Related Gene.

**Table 4:** Inhibition of fatty acid synthesis in cancer by plant extracts.

Plant extract	Cancer Type	Cancer cell lines	Action	References
*Allium cepa* L.	Breast	MDA-MB-231	Apoptotic effects via inhibiting FASN activity	[71]
*Vitis vinifera* L.	Breast	MCF-7, SKBR-3	Induced late apoptosis/necrosis by lowering FASN level	[77]
*Taraxacum mongolicum*	Breast	MDA-MB-231, MDA-MB-468	Interfere with unsaturated fatty acids metabolism via down-regulating the CHKA expression and inhibiting PI3K/AKT/SREBP/FADS2	[18]
*Ocimum minimum* L.	Breast	MDA-MB-231, MDA-MB-468	Cytotoxic, proapoptotic, and prooxidant effects via suppression of FASN	[78]
*Hippophae rhamnoides* L.	Breast	MDA-MB-231	Reduced cell viability and induced cell apoptosis via inhibition of FASN activity	[73]
